# Metabolic Profiling of Chicken Embryos Exposed to Perfluorooctanoic Acid (PFOA) and Agonists to Peroxisome Proliferator-Activated Receptors

**DOI:** 10.1371/journal.pone.0143780

**Published:** 2015-12-01

**Authors:** Anna Mattsson, Anna Kärrman, Rui Pinto, Björn Brunström

**Affiliations:** 1 Department of Environmental Toxicology, Evolutionary Biology Centre, Uppsala University, Uppsala, Sweden; 2 School of Science and Technology, Örebro University, Örebro, Sweden; 3 Computational Life Science Cluster (CLiC), Chemistry department (KBC) - Umeå University, Umeå, Sweden; 4 Bioinformatics Infrastructure for Life Sciences, Sweden; National Research Council of Italy, ITALY

## Abstract

Untargeted metabolic profiling of body fluids in experimental animals and humans exposed to chemicals may reveal early signs of toxicity and indicate toxicity pathways. Avian embryos develop separately from their mothers, which gives unique possibilities to study effects of chemicals during embryo development with minimal confounding factors from the mother. In this study we explored blood plasma and allantoic fluid from chicken embryos as matrices for revealing metabolic changes caused by exposure to chemicals during embryonic development. Embryos were exposed via egg injection on day 7 to the environmental pollutant perfluorooctanoic acid (PFOA), and effects on the metabolic profile on day 12 were compared with those caused by GW7647 and rosiglitazone, which are selective agonists to peroxisome-proliferator activated receptor α (PPARα) and PPARγ, respectively. Analysis of the metabolite concentrations from allantoic fluid by Orthogonal Partial Least Squares Discriminant Analysis (OPLS-DA) showed clear separation between the embryos exposed to GW7647, rosiglitazone, and vehicle control, respectively. In blood plasma only GW7647 caused a significant effect on the metabolic profile. PFOA induced embryo mortality and increased relative liver weight at the highest dose. Sublethal doses of PFOA did not significantly affect the metabolic profile in either matrix, although single metabolites appeared to be altered. Neonatal mortality by PFOA in the mouse has been suggested to be mediated via activation of PPARα. However, we found no similarity in the metabolite profile of chicken embryos exposed to PFOA with those of embryos exposed to PPAR agonists. This indicates that PFOA does not activate PPAR pathways in our model at concentrations in eggs and embryos well above those found in wild birds. The present study suggests that allantoic fluid and plasma from chicken embryos are useful and complementary matrices for exploring effects on the metabolic profile resulting from chemical exposure during embryonic development.

## Introduction

Metabolomics is an untargeted profiling approach where hundreds to thousands of metabolites with a high diversity of chemical structures are measured in a body fluid, tissue, or whole individual. The analytes are primarily small endogenous molecules, such as carbohydrates, amino acids, lipids and their derivatives. Metabolic profiling may provide information about changes in the biochemical/physiological status of an organism caused by environmental factors. Metabolomics is increasingly applied in the field of toxicology and has been used to investigate toxicity pathways, find associated mechanisms, and identify biomarkers of a certain exposure or endpoint. Metabolic profiling may also reveal effects not manifested as overt toxicity. The metabolomics technologies and their uses in toxicology and safety assessment have recently been reviewed by Ramirez et al. [1] and Bouhifd et al. [2].

The embryonated chicken egg provides a convenient model for exploring effects of compounds on embryonic development. Avian embryos develop separately from their mothers and, by contrast to exposure in mammalian models, chicken eggs can be injected with a defined dose of test substance without maternal exposure and thus without confounding factors such as maternal toxicity, maternal care or litter effects. Consequently, the single embryo in an egg can be considered a statistical unit in experimental studies. A further advantage when using chicken embryos for metabolic profiling is that embryonic blood and allantoic fluid are easily collected. The allantois is an extraembryonic membrane/sac that functions as a respiratory organ, a repository for urine and metabolic waste, and possibly also as a depot for various endogenous compounds [3, 4].

The perfluoroalkyl acids (PFAAs) comprise a large group of man-made organic chemicals consisting of a fluorinated carbon backbone with a charged functional group in one end (e.g. carboxylate, sulfonate, or phosphonate). The two most widely known PFAAs are perfluorooctanoic acid (PFOA) and perfluorooctane sulfonic acid (PFOS). PFAAs are widely used for instance in fire-fighting foams, in hydraulic fluids, as surfactants, and as surface protecting agents for textiles, paper and food packaging [5–7]. Due to extreme resistance to degradation and accumulation in humans and ecosystems globally these compounds have raised concern regarding possible adverse health effects in humans and wildlife [8–10].

In various mammalian models, PFOA has been associated with developmental effects, hepatic toxicity, tumors in specific organs, weight loss, and immunotoxicity, as well as altered levels of serum triacylglycerols and cholesterol, as reviewed previously [11–13]. Early life stages may be particularly sensitive and developmental exposure to PFOA and other PFAAs has been shown to reduce growth, delay development, cause neonatal death, impair mammary gland development and alter behaviors [12, 14–17]. In humans, PFOA and many other PFAAs are found in fetal cord blood [18–20] and in breast milk [21, 22] and can consequently be transferred from mother to fetus and suckling offspring. It was recently concluded that PFOA is “known to be toxic” to human reproduction based on evidence of decreased fetal growth in human and other mammalian species [23]. In birds and other oviparous species, PFAAs are excreted into the eggs and consequently the embryos are exposed during the whole developmental period *in ovo* [24, 25]. There is also some evidence from egg exposure studies in chicken that PFOA may cause embryo mortality [26, 27], reduce hatching success [28], cause cardiotoxicity [26], and induce neurobehavioral alterations [29]. Despite that the toxic properties of PFOA and some other fluorinated compounds have been extensively studied, their modes of action have not been fully elucidated.

PFAAs are structurally similar to natural fatty acids and some PFAAs have been shown to activate peroxisome proliferator-activated receptors (PPARs) [30, 31]. PPARs, of which the three subtypes α, β/δ, γ are known, are fatty acid-activated nuclear transcription factors that regulate genes involved in various functions, including lipid metabolism, energy homeostasis, cell differentiation and inflammation, as reviewed elsewhere [32, 33]. PFOA binds and activates human and rodent PPARα and to some degree PPARγ [30, 34, 35]. There is strong evidence that the hepatic effects induced by PFOA in rodents are largely mediated by activation of PPARα [36, 37]. Studies with knock-out mice showed that postnatal mortality in mouse could be mediated via mouse PPARα but not via a humanized form of the receptor, suggesting species differences in response to receptor binding [38, 39]. However, modest activation of PPARα during prenatal development using the agonists clofibrate and Wy14,643 caused no postnatal mortality in mouse [40]. In chicken, cardiac effects induced by embryonic PFOA exposure could partly be reproduced by the PPARα agonist Wy 14,643 [41]. Thus, it is uncertain to what extent effects of PFOA and other PFAAs in various species are due to PPAR activation.

The overall objective with this study was to explore blood plasma and allantoic fluid from chicken embryos as matrices for revealing metabolic changes caused by embryonic exposure to xenobiotics. The specific aims were 1) to explore whether embryonic exposure to PFOA at sublethal doses alters metabolite concentrations in allantoic fluid and blood plasma, and 2) to investigate whether PFOA causes metabolic changes similar to those caused by activation of PPARα or PPARγ by the selective agonists GW7647 and rosiglitazone, respectively. Our results suggest that blood plasma and allantoic fluid from chicken embryos are useful and complementary matrices for revealing metabolic changes following activation of PPARs. Sublethal doses of PFOA caused remarkably little effects on the metabolic profiles of the embryos and we found no evidence of PPAR activation by PFOA.

## Materials and Methods

### Exposure chemicals

Embryos were exposed to GW7647 (>99% purity; CAS: 265129-71-3; Tocris Bioscience, Minneapolis, MN, USA), rosiglitazone (≥98% purity; CAS: 122320-73-4; Sigma-Aldrich, St. Louis, MO, USA) or PFOA (free acid, ≥96% purity, CAS: 335-67-1; Sigma-Aldrich). Exposure solutions were prepared by dissolving the test substances in dimethyl sulfoxide (DMSO).

### *In ovo* exposure

The experimental procedures in this study were approved by the Uppsala Ethical Committee for Research on Animals (Permit number C 211/12). Fertilized chicken eggs (*Gallus gallus domesticus*) were obtained from a local breeder (Ova Production AB, Vittinge, Sweden). The egg weights were 60 ± 4 g (mean and standard deviation). The eggs were incubated at 37.5°C and 60% relative humidity with automatic turning every six hours. The day the eggs were placed in the incubator was defined as embryonic day zero (E0). Chicken embryos were exposed to test substances by air sac injection in eggs incubated for seven days (E7). At this stage, the extraembryonic allantoic blood vessel network has grown to cover the inner shell membrane beneath the air sac and the injected substance will come in direct contact with the vascular network. The exposures were performed as follows: Unfertilized eggs and poorly developing embryos were recognized by candling and were sorted out. Eggs with embryos of normal size were collected and randomly distributed into the different treatment groups. On E7, the blunt end of each egg was wiped with alcohol and a small hole was drilled through the shell above the air sac. The dissolved test substance (20 μl) was deposited onto the inner shell membrane in the air sac and the hole was sealed with melted paraffin wax. Following injection, eggs were held horizontally and rotated for a few seconds to allow dispersal over the inner shell membrane in the air sac. Each egg was marked with a number specifying the treatment group and was placed at a random location in the incubator.

The doses were 150 μg/egg (approximately 2.5 mg/kg egg) for GW7647 and rosiglitazone and 50, 250 or 1000 μg/egg for PFOA (approximately 0.83, 4.2 and 18 mg/kg egg, respectively). Controls were injected with 20 μL vehicle only (DMSO). Due to the large number of embryos required, the experiment was divided into four replicate experiments (replicate A-D) performed the same way but on separate occasions. The number of exposed embryos was 10–15 for each treatment group and replicate. The total number of exposed embryos in a group is given in Table 1.

**Table 1:** Injected doses, number of injected eggs, and number of samples of allantoic fluid and blood plasma subjected to metabolic profiling. Chicken eggs were injected on E7 and embryos were sampled on E12.

Group	Dose		No of eggs	Allantoic fluid		Blood plasma	
	(μg/egg)	(mg/kg egg)		f	m	f	m
Control	0	0	40	9	11	9	11
GW7647	150	2.5	40	8	11	9	11
Rosiglitazone	150	2.5	40	10	13	9	13
PFOA 50	50	0.83	40	11	7	11	7
PFOA 250	250	4.2	40	11	7	11	9
PFOA 1000	1000	18	54	-	-	-	-
Ʃ			210	49	49	49	51

### Embryo dissections and sampling

Embryos were dissected and sampled on E12. The treatment group was concealed to the one carrying out embryo dissections and the order of dissection of the embryos was randomized. The embryos were examined for viability and gross morphology, e.g. appearance of the liver. The sex was determined by the presence of a pair of testes or an ovary.

A subset of randomly chosen embryos from each group (except the 1000 μg/egg group) was sampled for metabolomic analysis of allantoic fluid and blood plasma. Samples of allantoic fluid and blood were collected before decapitation and dissection of the embryo as described below. The egg shell was removed above the air cell without disrupting the inner shell membrane. Allantoic fluid was collected by gently inserting a needle through the inner shell membrane and into the allantoic sac and then aspiring the fluid with a syringe. The samples were placed in microtubes on ice during sampling and then stored at -70°C until processed for metabolomic analysis. Blood was collected from the allantoic artery, just proximal to its first branch. The artery was gently cleaned from membranes, lifted with a small spoon and then punctured with a needle. Blood flowing into the spoon was immediately collected with heparinized capillaries. Blood was stored in heparinized microtubes on ice during sampling and was then centrifuged at a speed of 1300g for 10 min at 4°C. Plasma was collected and stored at -70°C. The embryos were killed by decapitation and dissected immediately following blood sampling.

Embryos and livers were weighed and the liver-somatic index was calculated (100 x liver weight/body weight). Livers were collected from the control group and from each of the PFOA groups for determination of PFOA concentrations. These livers were stored at -20°C and later extracted and analyzed as described below. Four females and four males from each PFOA group (except the 1000 μg/egg group) and from the control group were also randomly chosen for evaluation of signs of overt liver toxicity using liver histology (method described in S1 Methods).

The numbers of injected eggs and embryos used for metabolic profiling are given in Table 1. The numbers of embryos used for other endpoints or measurements are given in corresponding figures. For metabolomics, all replicates were represented with at least four individuals in each treatment group, except in the group exposed to 50 μg PFOA/egg in which replicate D was represented with two individuals. Due to high mortality rate in the group exposed to 1000 μg PFOA/egg, this group was not subjected to metabolomics, analysis of plasma lipids or liver histology, and only five embryos were weighed and examined for gross morphological changes. Liver concentrations of PFOA were determined in all PFOA-exposed groups and in controls.

### Plasma lipids

Total cholesterol, low-density lipoprotein (LDL) cholesterol, high-density lipoprotein (HDL) cholesterol, and triacylglycerols in plasma were analyzed with an Architect c8000 (Abbott Diagnostics, Abbott Park, IL, USA), using a photometric method, at the department of Clinical Chemistry and Pharmacology, Uppsala University Hospital, Sweden. The laboratory is accredited according to EN/ISO 15189 medical laboratory requirements. A total of 80 samples were analyzed; control: 7 females and 8 males; GW7647: 7 females and 8 males; rosiglitazone: 5 females and 12 males; 50 μg PFOA/egg: 11 females and 6 males; 250 μg PFOA/egg: 10 females and 6 males. All four replicate experiments were represented in each exposure group.

### Determination of PFOA in liver and plasma

The concentration of PFOA was analyzed in liver and blood plasma from control embryos and embryos exposed to PFOA (50, 250 and 1000 μg/egg). Five individual livers from each PFOA-treated group were analyzed. Very low PFOA concentrations were anticipated in the controls and therefore the amount of tissue was increased by pooling 2–3 livers, and five such pools were analyzed. Four plasma samples (50 μL) were analyzed from each group except the group exposed to 1000 μg PFOA/egg for which three samples were analyzed. Extraction blanks were included for each batch. The analyzed amount of liver homogenate was 100 mg for controls and 50 mg for PFOA-exposed embryos. Livers were homogenized using the bead mill Bullet Blender^®^ Storm 24 (Next Advance, Inc. New York, USA) with stainless steel beads at speed 8 for 3 min. Plasma and liver samples were spiked with 50 ng of internal standard (^13^C_4_-PFOA, Wellington, Guelph, Canada). Five milliliters of acetonitrile (Labscan, Gliwice, Poland) was added to the samples followed by vortex mixing, sonication (20 min), and centrifugation (10 000 g, 5 min). Extraction was performed twice for livers and once for plasma. One mL of supernatant from the extracts was transferred to a tube prepared with 50 mg ENVI-Carb^™^ 20/400 mesh (Supelco, Bellefonte, PA, USA) and 100 μL glacial acetic acid (Sigma-Aldrich). The carbon suspension was vortex mixed and then filtrated using a 0.2-μm GHP filter (Pall, East Hills, NY, USA). Filtrate (0.4 mL) was mixed with 2 ng of the recovery standard (^13^C_8_-PFOA, Wellington Laboratories) and 0.6 mL 2 mM ammonium acetate was added. Analysis was performed using an Acquity UPLC coupled to a Xevo-TQS MS/MS (Waters Corporation, Milford, MA, USA) as described previously [27]. The recovery standard ^13^C_8_-PFOA was added before injection to assess the recoveries of the internal standard ^13^C_4_-PFOA. The recoveries ranged between 75 and 94% (average 82%) for plasma and between 64 and 119% (average 84%) for liver.

### GC-MS and LC-MS

Metabolites in allantoic fluid and blood plasma were analyzed using gas chromatography-mass spectrometry (GC-MS) and liquid chromatography-mass spectrometry (LC-MS) as described in S1 Methods. Prior to GC-MS and LC-MS analysis, allantoic fluid (100 μL) and blood plasma (50 μL) were extracted. To each sample, 900 μL (allantoic fluid) or 450 μL (blood plasma) of extraction buffer (90/10 v/v methanol:water) including all internal standards were added. Standards for GC-MS were proline, succinic acid, glutamic acid, salicylic acid, alpha-ketoglutarate, putrescine, myristic acid, hexadecanoic acid, cholesterol, and sucrose. Standards for LC-MS were Val-Tyr-Val, Leu-enkephalin, sulfadimethoxine and reserpine. Samples were homogenized by shaking in a bead mill at a frequency of 30 Hz for 3 minutes and proteins were precipitated at +4°C on ice. The samples were centrifuged at +4°C, 14000 rpm, for 10 min. Then, 200 μL supernatant was transferred to a GC microvial and 200 μL was transferred to an LC microvial, and solvents were evaporated. Samples from the four replicate experiments were analyzed using in chronological order and within each replicate experiment the run order of the individual samples was randomized.

### Data processing and statistical analysis

The software GraphPad Prism version 5 (GraphPad Software Inc, San Diego, CA, USA) was used for univariate and bivariate statistics. Mortality rates were analyzed with Fisher’s exact test. Body weight, liver-somatic index and concentrations of plasma lipids were initially analyzed using two-way analysis of variance (two-way ANOVA) with treatment and sex (or treatment and replicate) as independent variables. Since these variables showed no differences related to sex or replicate they were re-analyzed using one-way ANOVA followed by Dunnett’s multiple comparison test where all treatment groups were compared to the control group. Metabolites were selected by multivariate methods as described below and differences in concentrations (treatment groups vs control group as well as females vs males) were then tested using Student’s *t*-test. The results of these *t*-tests on metabolite concentrations were used in an exploratory way to find candidate metabolites and for that reason the p-values were not corrected for multiple comparisons.

Multivariate statistical analyses of metabolite profiles were performed using the software package SIMCA-P version 13.0 (Umetrics, Umeå, Sweden). Data was centered and scaled to variance 1 (UV scaling) prior to modelling. To reduce unintended systematic variation caused by sample processing and instrument drift, GC-MS and LC-MS metabolite amounts (expressed as the area under the chromatographic peak) were normalized by Orthogonal Partial Least Squares analysis (O2PLS) prior to multivariate data analysis. O2PLS finds common (predictive) and unique (orthogonal) variation in two data matrices. If applied to find the common variation between the data matrix containing the measured metabolite concentrations and a matrix containing only the internal standards, the only common (predictive) variation expected is due to the unintended systematic variation. By deleting the contribution of the predictive latent variables, that variation can be removed from the data. Models obtained after this normalization contain in general less latent variables, and this simplification helps when deciding on the number of latent variables to be used. Following normalization, GC-MS and LC-MS data were analyzed together. Initially, a global Principal Component Analysis (PCA) was performed to get an overview of groups and trends and to identify outliers. Projections to latent structures by means of Orthogonal Partial Least Squares Discriminant Analysis (OPLS-DA) was used to model the relationship between the X-matrix (metabolite concentrations) and the Y matrix (class, i.e. exposure group or sex), and to find x-variables that were related to class belonging. OPLS-DA is a supervised prediction and regression method that rotates the projection plane to find the maximum covariance between the data and the class assignment. All groups were first analyzed together in a 5-class OPLS-DA and then each exposure group was analyzed against the control group in a 2-class OPLS-DA. Sex differences were analyzed both using the whole data set and in each group separately using 2-class OPLS-DA, with females and males as the two classes. Only variables with 80% or more nonzero values were included in the statistical models. To avoid over-fitting the models, the number of extracted latent variables was kept to a minimum and new ones were only added as long as they were significant according to cross-validation and substantially increased the cross-validation term, Q2. The validity of each model was also checked using CV-ANOVA (ANOVA of the cross-validated residuals). A significance level of p < 0.05 was used. For each model, *R* ^*2*^ *X*, *R* ^*2*^ *Y and Q* ^*2*^ values were calculated, where *R* ^*2*^ *X* is a measure of the fraction of x variation modeled in the extracted latent variables, *R* ^*2*^ *Y is a* measure of the fraction of y variation modeled and *Q* ^*2*^ *is an estimate of the predictive ability of the model and is calculated by cross-validation*.

### Selection of candidate metabolites

For each 2-class OPLS-DA model (also non-significant ones), metabolites were ranked according to their VIP score (Variable Importance for the Projection). The VIP score reflects the variable’s contribution to the separation of the classes and can be used for selecting the major discriminating variables in an OPLS-DA model. The variables (metabolites) with the highest VIP scores in each model were selected and re-analyzed using *t*-tests as described above. For practical reasons we tested only the top 50 and top 20 metabolites from each treatment or sex model, respectively. Metabolites that differed significantly (p<0.05) between control and treatment groups(s) or between sexes were considered candidate metabolites. The acquired mass spectra and retention indices (GC-MS) or masses and retention times (LC-MS) of the top candidates were compared with those of entries in our in-house databases [42] (Swedish Metabolomics Centre). Since it was not the scope of this work to fully identify individual metabolites the acquired tentative identities were not further analyzed or confirmed.

## Results

Because of high mortality rate, the embryos exposed to the highest PFOA dose (1000 μg/egg) were not sampled or analyzed regarding changes in metabolite patterns, plasma lipids and liver histology. Five embryos in this group were weighed and examined for gross morphological changes. Liver concentrations of PFOA were determined in five embryos and plasma concentration of PFOA in three embryos.

### Mortality and morphology

Embryo mortality rate was low in the control group (1/40; 2.5%). In the group exposed to the highest PFOA dose (1000 μg/egg) the mortality rate was 35%, which was significantly higher than that in the control group (p<0.0001). The mortality rate at the 1000 μg PFOA/egg dose differed between the four replicates (40, 60, 33 and 7% in replicate A, B, C and D, respectively). The increased mortality rate was confirmed in a follow-up study where the mortality was 40% in the group exposed to 1000 μg PFOA/egg and 12% in the control group (N = 25 in each group; p<0.05). In the other exposure groups the mortality rate was low and did not differ significantly from that of the control group (Table 1).

In the group exposed to the highest PFOA dose, body- and liver weights were determined for only five individuals, all from replicate A (as shown in Table G in S1 Tables). The liver-somatic index was significantly increased among these individuals, both when compared to the controls in replicate A and when compared to all controls (means were 2.0 ± 0.2 vs 1.7 ± 0.2 and p<0.01 in both cases, Table 2). Body weight and liver-somatic index were not affected by the other treatments (Table 2) and did not differ between sexes or replicates (the data is found in Table G in S1 Tables).

**Table 2:** Mortality rate, body weight and liver-somatic index. Chicken embryos were treated with various compounds by *in ovo* injection on E7 and were sampled on E12.

	Mortality rate		Body weight (g)		Liver-somatic index (%)	
	Ratio	%	Mean ± SD	N	Mean ± SD	N
Control	1/40	2.5	4.4 ± 0.3	24	1.7 ± 0.2	24
GW7647	0/40	0	4.2 ± 0.6	22	1.7 ± 0.1	22
Rosiglitazone	2/40	5	4.3 ± 0.4	29	1.7 ± 0.2	27
PFOA 50	2/40	5	4.2 ± 0.5	21	1.7 ± 0.1	21
PFOA 250	1/40	2.5	4.3 ± 0.4	27	1.7 ± 0.2	26
PFOA 1000^a^	19/54	35***	4.6 ± 0.4	5	2.0 ± 0.2**	5

N, number of embryos; ND, not determined; SD, standard deviation; Control eggs were injected with the vehicle, DMSO.

^a^Body weight and liver-somatic index were only determined for five embryos from replicate experiment A in this group;

***p<0.001;

**p<0.01.

Dissection and gross examination of the embryos revealed no abnormalities other than occasional white spots on the liver. These occurred at a frequency of 5–19% across all groups (13% in the control group). The spots were generally few and small and were located to the edges or apex of the liver lobes. However, some embryos showed larger affected areas on the liver. Livers from eight individuals from each PFOA group (except 1000 μg/egg) without any macroscopically visible abnormalities were subjected to crude histological examination. There were no obvious cases of disorganized tissue structure, invasion by leucocytes, or necrotic/apoptotic cells in any groups. The liver sections varied in their degree of intracytoplasmic vacuolization within all groups, including the control, but there was no correlation with treatment. Even though no treatment-related changes were observed in the histological sections, we cannot exclude occurrence of subtle histological changes.

### PFOA concentrations in liver and plasma

**Fig 1:**
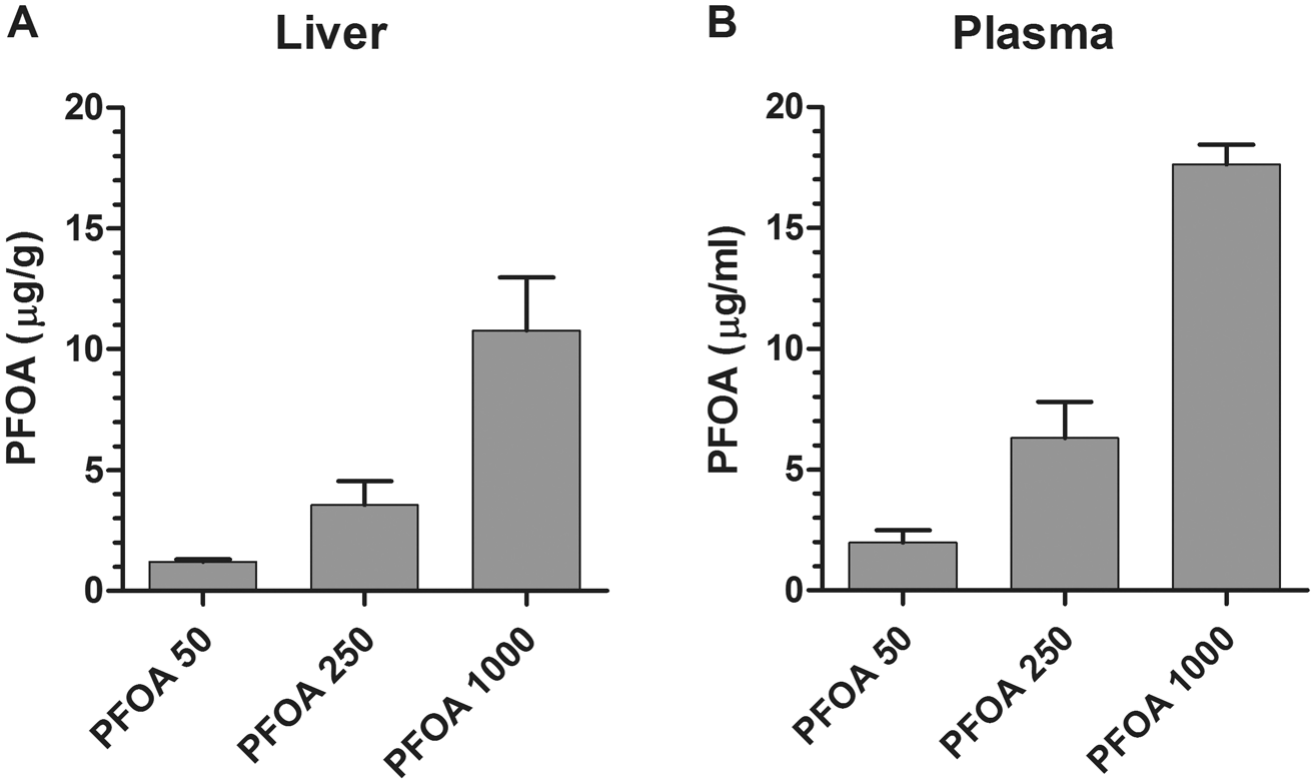
PFOA concentration (mean + SD) in (A) liver and (B) plasma. Chicken embryos were treated with PFOA (50, 250 or 1000 μg/egg) by *in ovo* injection on E7 and were sampled on E12. Liver PFOA concentration was analyzed in five individuals per group (mixed sexes), and plasma PFOA concentration was analyzed in four (50 and 250 μg PFOA/egg) or three (1000 μg PFOA/egg) individuals. Concentrations of PFOA in the control samples were similar to those in the procedural blanks.

The concentrations of PFOA in liver and plasma from PFOA-exposed embryos are shown in Fig 1. The present results and those from a previous study [27] showed no indication of sex differences in tissue concentration of PFOA in chicken embryos following egg injection and therefore the sexes were combined. The mean PFOA concentrations were 1.1, 3.6 and 11 μg/g wet weight in liver and 2.0, 6.3 and 18 μg/mL in plasma from embryos exposed to 50, 250 and 1000 μg PFOA/egg, respectively. Low levels of PFOA were detected in both controls and procedural blanks. The concentrations in controls were 0.07 ± 0.04 μg/g in liver and 0.3 ± 0.3 μg/mL in plasma. The levels measured in the procedural blanks corresponded to 80–290% and 100–140% of the control means in liver and plasma, respectively. Procedural blanks do not contain any tissue sample and the PFOA in the blanks is therefore attributed solely to materials and solvents added during sample processing. The similar levels in the control samples and in the procedural blanks indicate that the low concentrations of PFOA in controls are most likely attributed to contamination during sample preparation.

### Plasma lipids

Analysis with two-way-ANOVA revealed no sex differences in plasma lipid concentrations and therefore females and males were analyzed together. HDL-cholesterol concentration was slightly higher in the group exposed to GW7647 compared to the control (p<0.05), but was not affected by rosiglitazone or PFOA treatment (Table 3). Total cholesterol, LDL-cholesterol, the LDL/HDL ratio, and triacylglycerol concentrations were not significantly affected by any treatment.

**Table 3:** Concentrations of lipids (mM; mean ± standard deviation) in plasma. Chicken embryos were treated with various compounds by *in ovo* injection on E7 and were sampled on E12.

	N	Total C^a^	LDL-C^b^	HDL-C^c^	LDL/HDL	TAG^d^
Control	15	5.2 ± 0.6	1.5 ± 0.2	0.58 ± 0.04	2.6 ± 0.4	5.0 ± 1.0
GW7647	15	5.5 ± 0.3	1.5 ± 0.2	0.63 ± 0.05*	2.4 ± 0.4	4.9 ± 0.7
Rosiglitazone	17	5.2 ± 0.4	1.5 ± 0.2	0.60± 0.06	2.5 ± 0.4	5.4 ± 0.8
PFOA 50	17	5.2 ± 0.8	1.5 ± 0.2	0.59 ± 0.06	2.5 ± 0.3	5.0 ± 0.8
PFOA 250	16	5.0 ± 0.5	1.5 ± 0.3	0.57 ± 0.05	2.6 ± 0.5	5.0 ± 0.7

*P<0.05;

N, number of samples;

^a^Total cholesterol;

^b^Low-density lipoprotein cholesterol;

^c^High-density lipoprotein cholesterol;

^d^ Triacylglycerols

### Metabolite profiling of allantoic fluid and blood plasma

**Fig 2:**
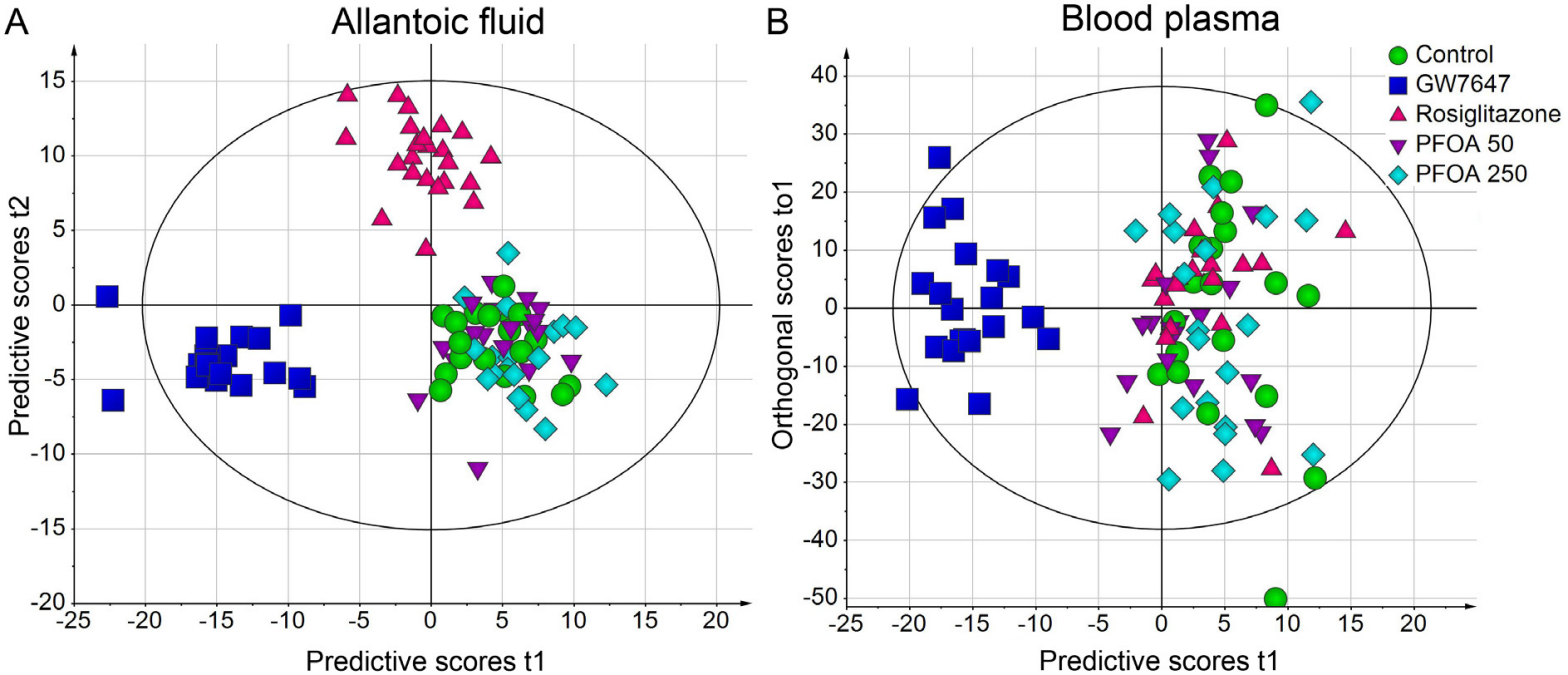
OPLS-DA score scatter plots of metabolites in (A) allantoic fluid and (B) plasma.

In allantoic fluid, 332 unique features (presumptive metabolites) were found using GC-MS and 737 were found using LC-MS. In plasma, 428 unique features were found with GC-MS and 1931 with LC-MS. Differences in metabolite pattern between the different exposure groups were analyzed using multivariate statistical analysis and visualized in score and loading plots. The metabolite concentration data was initially analyzed with PCA which did not reveal any separations between the groups or any gross outliers (data not shown). Analysis of allantoic fluid using the supervised method OPLS-DA revealed three completely separated clusters of observations (embryos); the two PFOA groups (50 and 250 μg PFOA/egg) clustered together with controls while embryos treated with GW7647 and rosiglitazone formed two distinct separate clusters (Fig 2A). When analyzing plasma, only GW7647-treated embryos clustered together separately from embryos of the other groups (Fig 2B).

The graphs show treatment-related differences in the metabolic profile in chicken embryos on E12 following treatment on E7 by *in ovo* injection. Classes correspond to treatments, i.e. vehicle control, GW7647, rosiglitazone and PFOA at 50 and 250 μg/egg. The ellipse shows a Hotelling's T2 95% confidence area. Axes show score vectors (*t*) of predictive latent variables 1 and 2 (A), and of predictive latent variable 1 and orthogonal latent variable 1 (B). The predictive latent variables show variation in X (metabolite concentrations) that is correlated with Y (treatment) whereas the orthogonal latent variable shows variation in X that is uncorrelated with Y. For plasma, only one significant predictive latent variable was obtained and the y-axis therefore shows orthogonal scores. OPLS-DA models were also computed for each treatment group vs the control group. Statistics for these 2-class models are shown in Table 4.

**Table 4:** Statistics of OPLS-DA models of metabolic profiles in chicken embryos treated with various compounds by *in ovo* injection on E7 and sampled on E12.

Classes	Matrix	A	N	R^2^X	R^2^Y	Q^2^	CV ANOVA
All treatments	AF	2 + 2	98	0.26	0.45	0.29	***
All treatments	Plasma	1 + 1	100	0.13	0.21	0.13	***
C vs GW7647	AF	1 + 1	39	0.17	0.96	0.72	***
C vs GW7647	Plasma	1 + 1	40	0.16	0.93	0.64	***
C vs rosiglitazone	AF	1 + 1	43	0.19	0.92	0.66	***
C vs rosiglitazone	Plasma	0 + 0	42	-	-	-	ns
C vs PFOA-50	AF	0 + 0	38	-	-	-	ns
C vs PFOA-50	Plasma	0 + 0	38	-	-	-	ns
C vs PFOA-250	AF	0 + 0	38	-	-	-	ns
C vs PFOA-250	Plasma	0 + 0	40	-	-	-	ns
Females vs males	AF	1+2	98	0.18	0.90	0.40	***
Females vs males	Plasma	1+2	100	0.17	0.89	0.37	***

AF, Allantoic fluid; A, Number of predictive + orthogonal latent variables; N, Number of samples;

***p<0.0001 in Cross Validation ANOVA; C, control.

Each treatment group was also modelled separately against the control group using a 2-class OPLS-DA in order to find candidate metabolites with potentially altered concentration. The characteristics of these models are summarized in Table 4. For GW7647, significant OPLS-DA models were obtained for both allantoic fluid and plasma. For rosiglitazone, a significant model was obtained for allantoic fluid but not for plasma. No significant models were obtained for PFOA, suggesting that PFOA caused no consistent effects on global metabolite profiles in either of the two studied fluids.

Candidate metabolites were selected from each OPLS-DA model based on their VIP-values and significance in *t*-test as described in the Materials and Methods section. These candidates can be found in Tables A and B in S1 Tables along with group means of relative concentrations, obtained masses (LC-MS), retention times, retention indices (GC-MS) and, when obtained, suggested molecular formulas (LC-MS) or tentative non-confirmed identities based on database matches. Although the PFOA groups did not separate from controls in the OPLS-DA models of global metabolite pattern, we found four metabolites in allantoic fluid and eight metabolites in plasma that were significantly affected by PFOA exposure (p<0.05 in *t*-test; Tables A and B in S1 Tables). Five of these 12 metabolites were also affected by either GW7647 or rosiglitazone, although only two were affected in the same direction. The identities of these metabolites were not determined.

**Fig 3:**
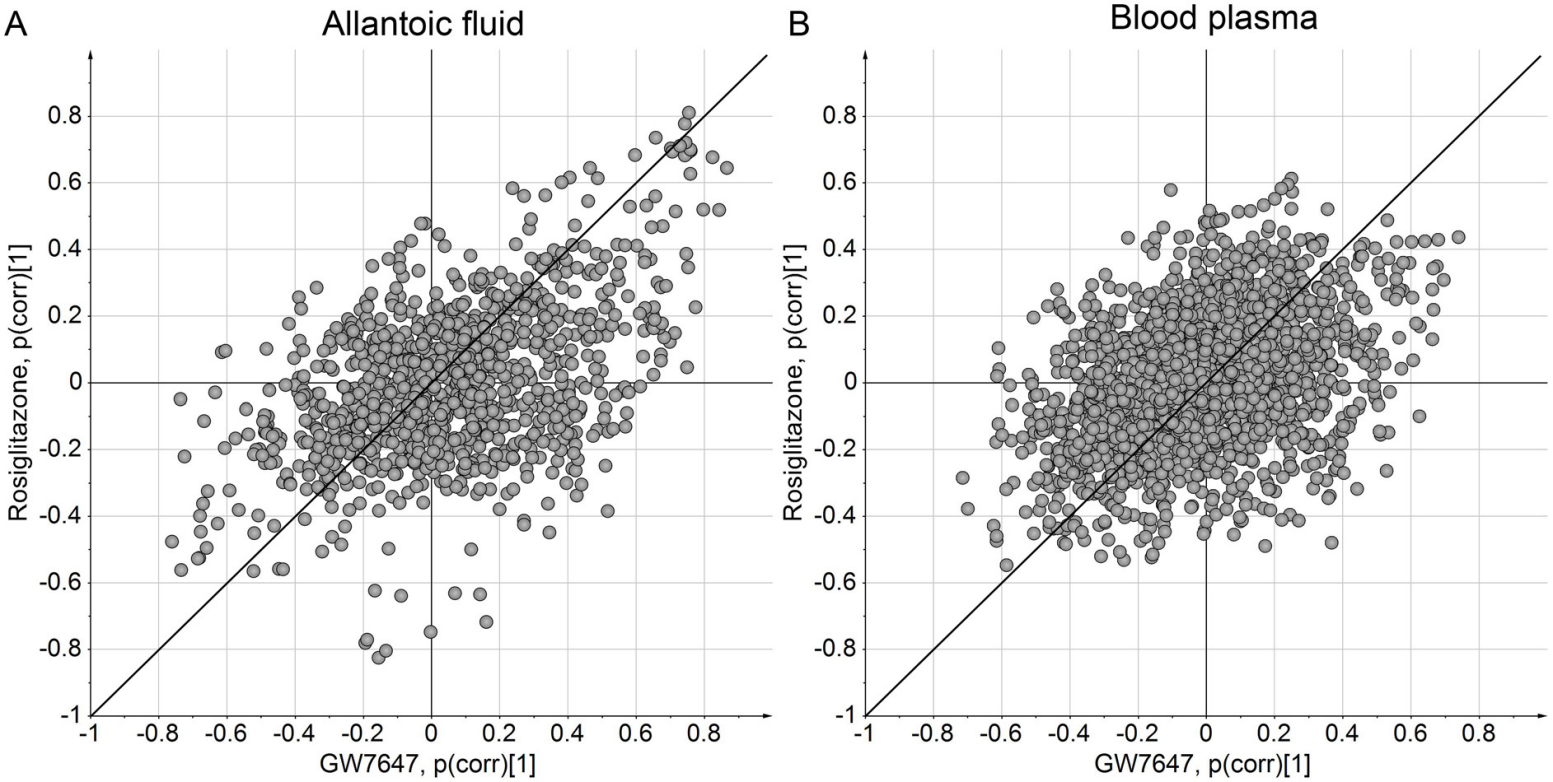
SUS-plots (Shared and Unique Structures) of GW7647 vs rosiglitazone OPLS-DA models. In the SUS-plot, the correlation from the predictive component of each of the two compared models is plotted against each other. The *x*-axis indicates change in concentration caused by GW7647 and the *y*-axis indicates change caused by rosiglitazone (upregulation at positive values and downregulation at negative values, compared to control). Thus, metabolites close to the diagonal running from the lower left corner to the upper right corner are affected by both GW7647 and rosiglitazone in a similar way, i.e. variables at the extremes of this diagonal are upregulated (top right) or downregulated (lower left) by both compounds. The plots show that many metabolites are affected in the same direction by the two compounds but there are also metabolites that are affected by only one of the compounds or affected in the opposite direction by the two compounds.

SUS-plots (Shared and Unique Structures) [43] were made by plotting the two OPLS-DA models “rosiglitazone vs control” and “GW7647 vs control” against each other (Fig 3). The SUS-plots visualize shared and unique changes caused by these compounds. The plots show that there are larger overall changes by GW7647 (*x*-axis) than by rosiglitazone (*y*-axis) in blood plasma, while in allantoic fluid the magnitude of change is similar for the two compounds. This is in agreement with the OPLS-DA-plots (Fig 2). The SUS-plots also show that there are more metabolites that are affected in the same direction (up- or downregulation) by these two compounds than in an opposite direction. In addition many metabolites are affected by only one of the compounds, for instance a cluster of metabolites that are downregulated by rosiglitazone in allantoic fluid.

**Fig 4:**
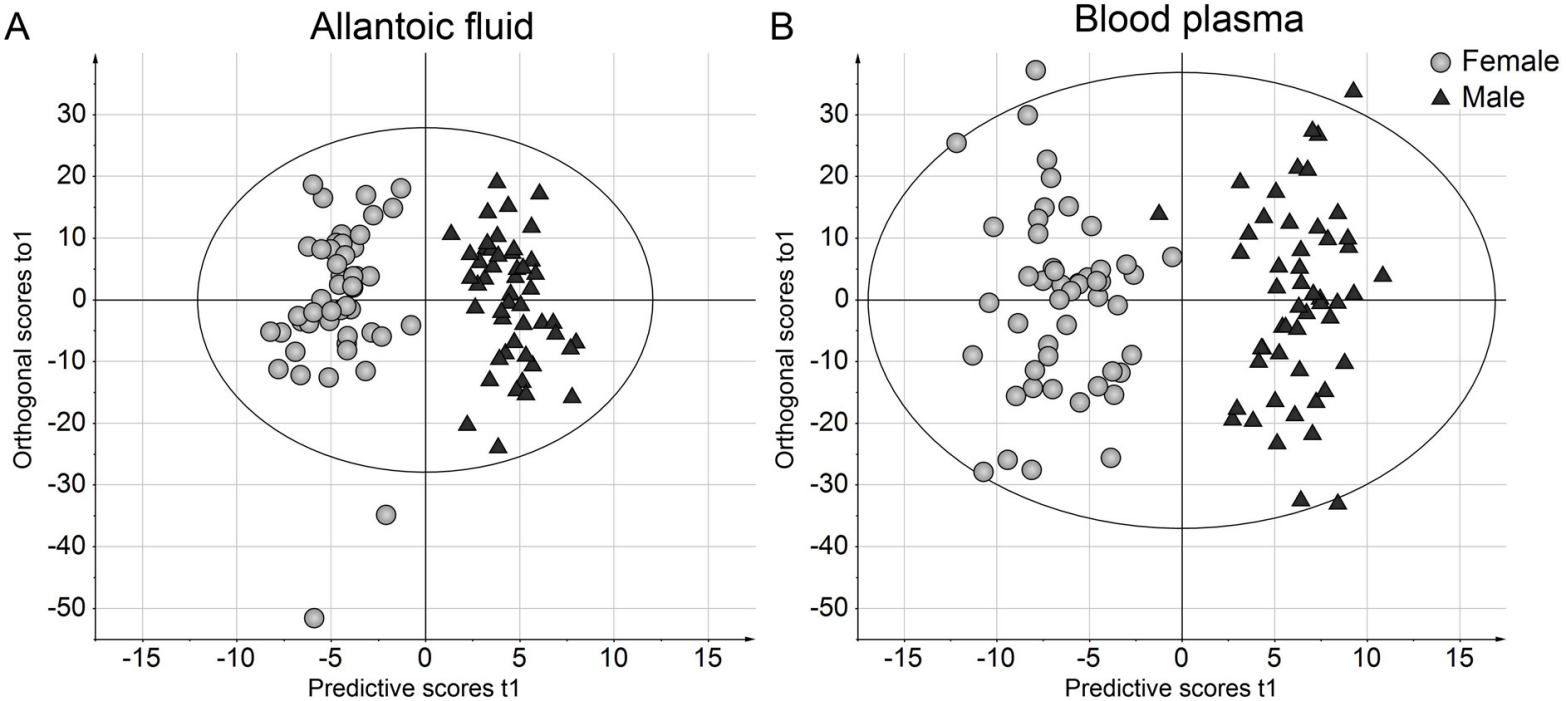
OPLS-DA score scatter plots of sex differences in the metabolic profile in (A) allantoic fluid and (B) plasma. Chicken embryos were treated by *in ovo* injection on E7 and were sampled on E12. Embryos from all studied treatment groups were assigned to two different classes based on their sex. The ellipse shows a Hotelling's T2 95% confidence area. Axes show score vectors (t) of predictive latent variable 1 and orthogonal latent variable 1. Model statistics are shown in Table 4.

We next evaluated whether chicken embryos exhibited sex differences in their metabolic profiles. First, all exposure groups were combined in one analysis. Class assignment according to sex resulted in significant OPLS-DA models for both allantoic fluid and plasma showing male and female clusters in score scatter plots (Fig 4 and Table 4). When sex difference was analyzed for each treatment group separately, a significant model was found for allantoic fluid from control embryos only. The 20 metabolites with the highest VIP value in the allantoic fluid and plasma models are listed in Tables A and B in S1 Tables. All of these metabolites showed significant sex difference when re-evaluated using Student’s *t*-test (p<0.001).

## Discussion

In the present study we used metabolic profiling (LC-MS and GC-MS) to explore exposure-related changes in the global profile of metabolite concentrations in allantoic fluid and blood plasma from chicken embryos following *in ovo* exposure to xenobiotics. The allantoic fluid and embryonic blood are easily accessible in birds, providing unique possibilities to study how compounds may perturb the endogenous metabolism of the embryo. The aim was to reveal any metabolic alterations by PFOA at sublethal doses in chicken embryos and to explore whether PFOA may cause changes similar to those caused by PPAR-activation. Both the PPARα-agonist GW7647 and the PPARγ-agonist rosiglitazone induced significant changes in the global metabolic profile whereas PFOA affected only a few metabolites. To our knowledge this is the first time allantoic fluid and blood plasma from chicken embryos have been used as matrices for evaluating effects by xenobiotics using untargeted metabolic profiling.

The allantoic fluid receives urine and metabolic waste throughout development in the chicken embryo. Alterations in the metabolic profile in allantoic fluid may therefore reflect treatment-induced changes in metabolic pathways at any time during the developmental period from treatment until sampling. Blood plasma on the other hand may provide a more instant picture of the metabolic status in the embryo at the time of sampling. Accordingly, changes in metabolite pattern were more evident in allantoic fluid than in blood plasma in rosiglitazone-treated embryos. The clustering of treatment groups and sexes were also “tighter” for allantoic fluid than for plasma suggesting that profiling of allantoic fluid is a robust approach with little individual variation within the respective classes.

The concentrations of endogenous metabolites in allantoic fluid, blood and amniotic fluid in the chicken egg and embryo have previously been studied in the context of regulation by hormones or stressors [44–47]. Feng et al. (2007) identified 61 metabolites and 26 chemical fragments in the allantoic fluid of 9-day-old chicken embryos using NMR (nuclear magnetic resonance) [48]. In the present study of 12-day-old chicken embryos, >300 features were detected in allantoic fluid using GC-MS and >700 were detected using LC-MS. For plasma the numbers were a bit higher (>400 and >1900, respectively). However, for most of these features the identity has not been revealed and as yet we do not know how many of these that represent unique metabolites.

The environmental contaminant PFOA caused little effects in the embryos besides increased mortality rate and increased liver-somatic index (N = 5) at the highest dose, i.e. 1000 μg/egg (~18 mg/kg egg). The two lower PFOA doses, 50 and 250 μg/egg (~0.83 and 4.2 mg/kg egg), caused no significant changes in mortality rate, body weight, liver-somatic index, concentrations of plasma triacylglycerols or cholesterol (LDL, HDL and total cholesterol), and there were no overt signs of histopathological changes in the liver.

There is some inconsistency between studies regarding the lowest PFOA dose that causes embryo mortality or reduces hatching success in chicken embryos. In studies where injection was done before start of incubation, increased mortality was reported at 2 mg/kg egg [26] and 5 mg/kg egg [28], while lack of effect on hatching success or mortality at doses up to 10 mg/kg egg was reported in another study [49]. We found no effect on survival at 4.2 mg/kg egg but increased mortality at 18 mg/kg egg, and thus the lowest observed effect level of 2 mg/kg [26] was lower than the no effect level in our study. It should be noted however that the embryos in the present study were exposed only from E7 to E12, and embryos may be more sensitive at earlier stages.

The increased liver weight found at the 1000 μg PFOA/egg dose is consistent with observations in mice [50] and salmon larvae [51]. Neither PFOA nor GW7647 caused any major changes in plasma lipid levels, suggesting that lipid concentrations in plasma may not be a sensitive indicator of PPARα-activation in chicken embryos following short-term exposure. In certain other species, however, PFOA has lipid-lowering effects that seem to be partly due to PPARα-activation. Oral exposure of adult mice to PFOA resulted in rapid reduction of body- and adipose tissue weight, increase in absolute and relative liver weight, and decrease in serum cholesterol and triacylglycerol levels [50, 52]. Decreased serum cholesterol and triacylglycerol levels have also been observed following perinatal exposure of mice to PFOA [53], and whole body levels of cholesterol were decreased in PFOA-exposed salmon larvae [51]. In humans, PFOA has on the other hand been associated with increased serum cholesterol and triacylglycerol levels at high environmental or occupational exposure [54–56] as well as at background exposure experienced by the general population [57]. However, the results from epidemiological studies are inconsistent [58] and whether there is causality between PFOS/PFOA exposure and increased serum cholesterol has been questioned [59].

Reasons for interspecies differences in response to PFOA may include differences in kinetics, in affinity for receptors, and in downstream effects from activation of receptors. For instance, while mouse PPARα mediated neonatal lethality in mice exposed to PFOA, this effect was not observed in mice expressing human PPARα [39]. The high levels of PFOA found in liver and plasma from PFOA-exposed embryos in the present study show that the substance was readily absorbed and was presumably available to various target organs/tissues. The activity of PFOA on the PPAR receptors or on other receptors has to the best of our knowledge not been studied in avian species, and it might well be that PFOA is not a very efficient PPAR-activator in the chicken. As discussed below, the metabolic profiling of PFOA-treated chicken embryos revealed no metabolic changes related to PPARα or PPARγ-activation. In a study by Strömqvist et al. [27], PFOA induced expression of the PPARα-regulated gene acylcoenzyme A oxidase in chicken embryos whereas other PPARα-regulated genes that were induced by GW7647 in the same study were unaffected by PFOA, suggesting a rather modest activation of PPARα by PFOA in chicken. On the other hand, exposure of chicken embryos to PFOA or the PPARα agonist Wy 14,643 cased overlapping, although not identical, effects on cardiotoxicity [41].

Our intention with doing metabolic profiling was to reveal alterations not related to mortality or other overt toxicity. Living embryos exposed to the highest PFOA dose (1000 μg/egg) were therefore not included in the metabolomic analyses. We performed metabolomic analyses of allantoic fluid and blood plasma in embryos of eggs injected with 50 and 250 μg PFOA/egg and found that PFOA caused little change in the metabolic profiles at these doses; the multivariate statistical models revealed no significant difference in global metabolic profile between the control group and the two analyzed PFOA groups. As shown in Fig 2A, embryos treated with the PPARα agonist GW7647 and the PPARγ agonist rosiglitazone form two completely separate clusters whereas controls and PFOA-treated embryos form a joint cluster based on metabolite concentrations in allantoic fluid. There was no significant difference in metabolic profile between PFOA-exposed embryos and controls even when each PFOA group was modelled separately against the control. Twelve individual metabolites were significantly affected (p<0.05, *t*-test) by PFOA at one or both doses; however it is possible that this was a chance finding as numerous metabolites were analyzed. Some of these twelve metabolites were also affected by either GW7647 or rosiglitazone but generally not in the same direction. Thus, there was no indication that PFOA would cause metabolic changes in chicken embryos through activation of PPARα or PPARγ. The identities of the metabolites affected by PFOA could not be revealed by searches in our in-house databases. Any effect of PFOA on the abundance of these metabolites should be confirmed in a further study; if results are consistent for some metabolites it would be worthwhile to make efforts to identify them. A possible biological significance may be elucidated if the affected metabolites can be associated with common metabolic pathways that may, or may not, be associated with previously observed morphological or behavioral changes. It should also be mentioned that many additional metabolites may well have been affected without our knowledge since no single analytical method (extraction method and platform) can cover all existing metabolites with their various characteristics.

It is possible that longer duration of exposure to PFOA, including both earlier and later developmental stages, would have resulted in significant effects on the metabolic profile in chicken embryos. The exposure period E7-E12 was chosen based on a pilot study in which exposure to GW7647 at E7-E12 induced selected PPARα-regulated genes in the liver at non-lethal doses, whereas exposure at E4-E14 induced PPARα-regulated genes only at doses causing increased mortality. Possibly, early exposure resulted in toxicity and mortality not related to PPARα-activation. It would be of interest to explore effects on the metabolome after longer exposure to PFOA, for instance covering early differentiation of gonads, liver and brain.

There are no in vivo studies on effects of PFOA on the metabolome in other species. However, the effects of PFOA on a human liver cell line have previously been investigated using a metabolomics approach together with transcriptomics and targeted analyses of selected genes and metabolites. They found perturbations in various metabolic pathways, especially lipid metabolism-related pathways. Effects were seen on levels of carnitine and acylcarnitines (involved in fatty acid oxidation), cholesterol (increased), nucleic acids, amino acids and some lipids [60].

The PFOA doses applied in the present study were higher than the levels found in eggs of wild birds [24, 25, 61–63] or in plasma from nestlings [64]. In great cormorant in lake Vänern in Sweden, median PFOA concentration in eggs was 4.0 ng/g and in embryo livers 1.7 ng/g. PFOS concentrations were more than two orders of magnitude higher. The injected doses of PFOA in the present study were approximately 0.83, 4.2 and 18 μg/g egg and these resulted in liver concentrations of 1.1, 3.6 and 11 μg/g wet weight and plasma concentrations of 2.0, 6.3 and 18 μg/mL in E12 chicken embryos. In humans, the concentrations of PFOA in serum or fetal cord blood are generally in the lower ng/mL-range [12, 18–20, 65], but occupationally exposed groups may experience much higher concentrations. For example, median blood levels in Swedish ski waxing technicians exposed to fluorinated wax fumes were 112 ng/mL compared with 2.5 ng/mL in the general Swedish population [66]. Exposed workers in a fluoropolymer manufacturing facility (Ohio, West Virginia) showed a median serum concentration of 775 ng/mL and non-occupationally exposed residents living in the community surrounding the facility had a median concentration of 329 ng/mL [67]. Serum levels of PFOA as high as 5100 ng/mL have been detected in individual retired fluorochemical production workers [68]; this concentration is within the range of the plasma concentrations of PFOA in the present study.

The distinct differences in metabolic patterns after activation of PPARα with GW7647 and PPARγ with rosiglitazone suggest that the metabolomics approach used in this study can successfully distinguish compounds that interact with either of these PPAR receptors. GW7647 is a thioisobutyric acid derivative that has been shown to be a potent and highly selective agonist for human PPARα in reporter cell lines [69, 70]. In a previous experiment it was shown that GW7647 significantly upregulated several PPARα-regulated genes in liver and kidney in chicken embryos, verifying that GW7647 indeed acts as a PPARα activator in chicken embryos [27]. Rosiglitazone is a thiazolidinedione and a potent agonist of human PPARγ; it shows no or little activation of PPARα or PPARδ in reporter cell assays [69, 71]. The weaker response to PPARγ activation compared with PPARα activation in the present study may reflect differences in physiological functions or importance of PPARα and PPARγ in the chicken embryo. However, it may also be a result of differences in potency or efficacy of the two model compounds used. In accordance with this, GW7647 was a more potent activator of transcription through human PPARα than rosiglitazone was through human PPARγ in a reporter cell assay (EC50 was 6 nM for GW7647 and 18 nM for rosiglitazone) [69]. In our study, many metabolites were affected in the same direction (up- or downregulation) by these two compounds and a smaller fraction was affected in the opposite direction (Fig 3). This indicates that GW7647 and rosiglitazone may also have some agonist activity on chicken PPARγ and PPARα, respectively, and/or that the pathways regulated by the two receptors partly overlap.

The metabolic pattern of both allantoic fluid and plasma in E12 chicken embryos showed marked sexual dimorphism when analyzed across all experimental groups. Morphological and biochemical sex differentiation of the gonads are evident already from E6 in the chicken embryo and during the developmental period in the present study (E7-E12) the male and the female embryos are exposed to different hormonal milieus [72]. This difference in hormonal milieu of the sexes is likely to result in differences in the respective metabolomes. Moreover, microarray studies have revealed sex differences in gene expression in chicken embryos even before gonadal sex differentiation [73, 74]. Consequently, the sex differences in metabolic profiles observed in the present study is biologically plausible.

## Conclusions

Untargeted metabolic profiling of allantoic fluid and blood plasma from chicken embryos proved to be applicable for revealing similarities and differences in effects caused by treatment with the PPARα agonist GW7647 and the PPARγ agonist rosiglitazone. However, the metabolic profiles of these fluids were not significantly changed by the environmental contaminant PFOA at plasma levels well above those generally found in wild birds as well as in humans. When comparing the metabolic profiles of the differently treated embryos, we found no evidence that PFOA activated PPARα or PPARγ. The effects from treatment with the different compounds on the metabolic profiles differed in allantoic fluid and plasma and our results suggest that allantoic fluid and plasma from chicken embryos are useful and complementary matrices for exploring effects on the metabolic profile from embryonic treatment with xenobiotics.

## Supporting Information

S1 Methods This file contains supporting information regarding experimental procedures of liver histology, GC-MS, and LC-MS. (DOCX)

S1 Tables Excel file containing supplementary Tables A-G. The tables contain the following data: Metabolite features in allantoic fluid affected by treatment or sex sorted by descending VIP-value **(Table A)**. Metabolite features in blood plasma affected by treatment or sex sorted by descending VIP-value **(Table B)**. Concentrations of all metabolic features measured in allantoic fluid and blood plasma **(Table C)**. PFOA concentrations in plasma from chicken embryos treated with vehicle control or PFOA at 50, 250 or 1000 μg/egg **(Table D)**. PFOA concentrations in liver from chicken embryos treated with vehicle control or PFOA at 50, 250 or 1000 μg/egg **(Table E)**. Concentrations of lipids (mM) in plasma **(Table F)**. Body weight, liver weight and liver-somatic index **(Table G)**. Chicken embryos were treated with vehicle, GW7647, rosiglitazone or PFOA by *in ovo* injection on E7 and were sampled on E12.
(XLSX)

## Abbreviations

ANOVA: analysis of variance
CV-ANOVA: Cross Validation ANOVA
DMSO: dimethyl sulfoxide
E7: embryonic day seven
GC: gas chromatography
HDL-C: high-density lipoprotein cholesterol
LC: liquid chromatography
LDL-C: low-density lipoprotein cholesterol
MS: mass spectrometry
O2PLS: Orthogonal Partial Least Squares
OPLS-DA: Orthogonal Partial Least Squares Discriminant Analysis
PCA: Principal Component Analysis
PFAAs: perfluoroalkyl acids
PFOA: Perfluorooctanoic acid
PFOS: perfluorooctane sulfonic acid
PPARs: peroxisome proliferator-activated receptors
SUS: Shared and Unique Structures
VIP: Variable Importance for the Projection
