# Supplementary material for: Metabolic Profiling of Chicken Embryos Exposed to Perfluorooctanoic Acid (PFOA) and Agonists to Peroxisome Proliferator-Activated Receptors
Source: PLoS One. 2015 Dec 1;10(12):e0143780. doi: 10.1371/journal.pone.0143780 (PMC4666608; doi:10.1371/journal.pone.0143780)
Supplement: S1 Methods — (DOCX) [file pone.0143780.s001.docx]

Supporting information: S1 Methods

Metabolic profiling of chicken embryos exposed to perfluorooctanoic acid (PFOA) and agonists to peroxisome proliferator-activated receptors

Anna Mattsson*^1^, Anna Kärrman^2^, Rui Pinto^3,4^, Björn Brunström^1^

^1^Department of Environmental Toxicology, Evolutionary Biology Centre, Uppsala University, Uppsala, Sweden.

^2^School of Science and Technology, Örebro University, Örebro, Sweden
^3^Computational Life Science Cluster (CLiC), Chemistry department (KBC) - Umeå University, Umeå, Sweden
^4^Bioinformatics Infrastructure for Life Sciences, Sweden.

This file contains supporting information regarding experimental procedures of:

# Liver histology

# Gas chromatography-mass spectrometry (GC-MS)

# Liquid chromatography-mass spectrometry (LC-MS)

# Liver histology

A piece of the liver was sampled and placed in phosphate buffered formalin (4% formaldehyde in 0.1 M phosphate buffer, pH 7.4; v/v). The liver pieces were dehydrated by treatment with ethanol in a series of increasing concentration (70%, 95%, and absolute ethanol; v/v) followed by soaking in xylene. The dehydrated liver tissue was embedded in Technovit 7100 (Heraeus Kulzer, Hanav, Germany) and six consecutive sections (2 µm) from the middle part of the liver were collected. The sections were mounted on Superfrost glass slides and stained with hematoxylin and eosin. Three sections from each liver were examined for overt signs of toxicity. First, the evaluator examined all sections with knowledge of their identities. Then all sections were re-evaluated several times with masked identities and notes were taken for each section. Evaluated features were general tissue structure, degree of vacuolization (graded 1-3), and the presence of leucocytes, necrotic cells and apoptotic cells. The notes for the treatment groups were then compared with those for the control group.

# Gas chromatography-mass spectrometry (GC-MS)

Metabolites were derivatized prior to GC-MS analysis. To each dry sample, 30 µL of methoxyamine (15 µg/µL in pyridine) were added. Samples were then vortexed for 10 minutes and incubated at 70 °C for 1 hour before letting the reaction proceed in room temperature for 16 hours. After adding 30 µL of N-methyl-N-trimethylsilyl-trifluoroacetamide (MSTFA containing 1% TMCS) the samples were vortexed and left to react for 1 hour in room temperature. Before analysis, 30 µL of methyl stearate (15 ng/µL in heptane) were added.

An aliquot of 1 µL of the derivatized sample was injected splitless by a CTC Combi Pal autosampler (CTC Analytics AG, Switzerland) into an Agilent 6890 gas chromatograph (Agilent Technologies Inc., Santa Clara, CA, USA) equipped with a 10 m x 0.18 mm fused silica capillary column with a chemically bonded 0.18 µm DB 5-MS UI stationary phase (J&W Scientific, Folsom, CA, USA). The injector temperature was 270 °C, the purge flow rate was 20 mL/min and the purge was turned on after 60 sec. The gas flow rate through the column was 1 mL/min, the column temperature was held at 70 °C for 2 min, then increased by 40 °C/min to 320 °C, and held there for 2 min. The column effluent was introduced into the ion source of a Pegasus III time-of-flight mass spectrometer, GC/TOFMS (Leco Corp., St Joseph, MI, USA). The transfer line and the ion source temperatures were 250 °C and 200 °C, respectively. Ions were generated by a 70 eV electron beam at an ionization current of 2.0 mA, and 30 spectra/sec were recorded in the mass range m/z 50 - 800. The acceleration voltage was turned on after a solvent delay of 150 sec. The detector voltage was 1500-2000 V.

All non-processed MS-files from the metabolic analysis were exported from the ChromaTOF software in NetCDF format to MATLAB™ R2011b (Mathworks, Natick, MA, USA), where all data pre-treatment procedures, such as base-line correction, chromatogram alignment, data compression and Hierarchical Multivariate Curve Resolution (H-MCR), were performed using custom scripts [[1](#_ENREF_1)]. Features, i.e. ions at a certain retention time, were identified by comparing the acquired mass spectra and retention indices with those of entries in inhouse mass spectra libraries at the Swedish Metabonomics Centre [[2](#_ENREF_2)].

# Liquid chromatography-mass spectrometry (LC-MS)

The samples were first analyzed in positive mode and then each sample was injected a second time and analyzed in negative mode. Liquid chromatography was performed on an Agilent 1290 Infinity UHPLC-system (Agilent Technologies). A 2 µL aliquot of each extracted sample was injected onto a 2.1 x 100 mm, 1.7 µm Kinetex C_18_ column (Phenomenex, Torrace, USA) held at 40 °C. The gradient elution buffers were A (H_2_O, 0.1% formic acid) and B (75/25 acetonitrile:2-propanol, 0.1% formic acid), and the flow-rate was 0.5 mL/min. The compounds were eluted with a linear gradient consisting of 1-20% B over 0-4 minutes, 20-40% of B over 4-6 minutes, 40-95% B over 6-9 minutes. The composition was held at 95% B for 4.5 minutes, and returned to 1% B at 14.5 minutes. The composition was kept at 1% B for a further 4.5 minutes before the next injection. The metabolites were detected with an Agilent 6550 Q-TOF mass spectrometer equipped with a jet stream electrospray ion source operating in positive or negative ion mode. The settings were kept identical between the modes, with exception of the capillary voltage. A reference interface was connected for accurate mass measurements; the reference ions purine (2 µM) and HP-0921 (Hexakis(1H, 1H, 3H-tetrafluoropropoxy)phosphazine) (2.5 µM) (Agilent Technologies) were infused directly into the MS at a flow rate of 0.05 mL/min for internal calibration, and the monitored ions were purine m/z 121.05 and m/z 119.03632; HP-0921 m/z 922.0098 and m/z 966.000725 for positive and negative mode respectively. The gas temperature was 150°C, the drying gas flow was 16 L/min and the nebulizer pressure was 35 psig. The sheath gas temp was 350 °C and the sheath gas flow was 11 L/min. The capillary voltage was 4000 V in positive ion mode, and 4000 V in negative ion mode. The nozzle voltage was 300 V. The fragmentor voltage was 380 V, the skimmer 45 V and the OCT 1 RF Vpp 750 V. The collision energy was set to 0 V. The m/z range was 70 - 1700, and data was collected in centroid mode with an acquisition rate of 4 scans/sec (1977 transients/spectrum). The diode array detector was set to scan the interval 190-640 nm with a step length of 2 nm and a slit width of 4 nm.

Mass feature extraction from the acquired data was performed using the MassHunter™ Qualitative Analysis software package, version B06.00 (Agilent Technologies). Extracted features were aligned and matched between samples using Mass Profiler Professional™ 12.5 (Agilent Technologies). Features were identified by comparing the acquired mass and retention time to those of entries in our inhouse database at the Swedish Metabonomics Centre.

References

1. Jonsson P, Johansson AI, Gullberg J, Trygg J, A J, Grung B, et al. High-throughput data analysis for detecting and identifying differences between samples in GC/MS-based metabolomic analyses. Anal Chem. 2005;77(17):5635-42.

2. Schauer N, Steinhauser D, Strelkov S, Schomburg D, Allison G, Moritz T, et al. GC-MS libraries for the rapid identification of metabolites in complex biological samples. FEBS Lett. 2005;579(6):1332-7.
